# Differential response of immune-related genes to peptidoglycan and lipoteichoic acid challenge *in vitro*

**DOI:** 10.14202/vetworld.2016.983-988

**Published:** 2016-09-17

**Authors:** Sourabh Sulabh, Bharat Bhushan, Manjit Panigrahi, Ankita Verma, Naseer Ahmad Baba, Pushpendra Kumar

**Affiliations:** Division of Animal Genetics, ICAR-Indian Veterinary Research Institute, Izatnagar, Bareilly - 243 122, Uttar Pradesh, India

**Keywords:** expression, lipoteichoic acid, mastitis, peptidoglycan, peripheral blood mononuclear cell

## Abstract

**Aim:**

To study the effect of *Staphylococcus aureus* cell wall antigens, peptidoglycan (PGN) and lipoteichoic acid (LTA) challenge on immune cells present in bovine peripheral blood mononuclear cells (PBMCs).

**Materials and Methods:**

In this study, efforts have been made to investigate the effects of three combinations (10+10, 20+20 and 30+30 μg/ml) of PGN and LTA obtained from *S. aureus*. These antigens were used to challenge the bovine PBMCs. After 6 h of incubation quantitative, real time-polymerase chain reaction was used to study toll-like receptor 2 (TLR-2) and major cytokine mRNA expression in bovine PBMC challenged with three different antigen blends.

**Results:**

The results indicated that mRNA level of interferon gamma is influenced by the expression of TLR-2 gene. Tumor necrosis factor-alpha (TNF-α), interleukin 10 (IL-10), and IL-8 genes showed a maximum response at a dose of 10 μg of PGN and 10 μg of LTA challenge per ml of culture medium. The outcome also suggests that both IL-10 and IL-8 followed the expression pattern of TNF-α.

**Conclusion:**

A dose of 10 μg of PGN and 10 μg of LTA per ml of culture medium was found to be most suitable for challenging PBMC.

## Introduction

Inflammation of the mammary gland and udder tissue is known as mastitis. The most pertinent cause of mastitis is the immune response that occurs due to the various bacterial sources invading the teat canal. The losses due to mastitis have been estimated to the tune of about 2 billion dollars in the USA and 526 million dollars in India [1]. Its prevalence is common even in very well-managed dairy farms. Bovine mastitis is most commonly caused by *Staphylococcus aureus, Streptococcus uberis*, and *Escherichia coli* [2]. Mastitis due to *S. aureus* infection is known to generally cause sub-clinical form of the infection which has a tendency to show resistance against antibiotic therapy [3]. Peptidoglycan (PGN) and lipoteichoic acid (LTA) present in the cell wall of Gram-positive bacteria, one of which is *S. aureus* have been recognized as a potent antigen having ability to stimulate the immune system of the host [4]. PGN and LTA act as pathogen-associated molecular patterns for pattern recognition receptors (PRRs) such as membrane protein and toll-like receptor 2 (TLR-2) [5]. Lymphocytes, monocytes, and macrophages serve as the effective regulators of inflammation and serve as the foremost line of the immune system against the invading microbes [6]. These cells have the capability to produce inflammatory conciliator such as cytokines and chemokines, which act by engaging other immune cells to the site of inflammation. Lymphocytes are present in the mammary gland secretions and play an important role in providing protection against mastitis causing organisms [7]. Various studies have recognized that the composition of the T-lymphocyte subpopulation showed susceptibility or resistance to intramammary infection [8]. Monocytes are known to produce a high amount of potent proinflammatory mediators. Macrophages are directly involved in phagocytosis of foreign bodies and also act as an important antigen presenting cell [9]. Their local recruitment and activity provide protection to the mammary gland from invading pathogens [10].

Although *S. aureus* vaccine development has not been successful either in humans or in animals, further insight and study of cytokine expression pattern may help in finding an appropriate solution. Evidence of the use of *S. aureus* ghost vaccine having only the outer envelope of the cell (containing PGN, LTA, and proteins) and also a separate experiment of injecting only PGN was found to induce protective immunity to a lethal challenge in experimental animals [11,12].

In this study, an attempt has been made to investigate the differential expression of genes in peripheral blood mononuclear cells (PBMCs) from crossbred cattle challenged with different doses of *S. aureus* antigens like PGN and LTA.

## Materials and Methods

### Ethical approval

Collection of blood required for the study was approved by the Institutional Animal Ethics Committee.

### Selection of animals and blood collection

Healthy crossbred cattle (50-75% inheritance from cattle breeds involving Holstein-Friesian, Jersey, and Brown Swiss with the native indigenous component stabilized in between 25% and 50% from Hariana cattle breed) maintained at cattle and buffalo farm, ICAR-Indian Veterinary Research Institute Izatnagar, in their 3^rd^ and 4^th^ parities were screened on the basis of the records of mastitis occurrence. Only those animals that were never affected by mastitis were selected for the study. To rule out the probability of sub-clinical mastitis, they were then examined for milk somatic cell count (SCC) and California mastitis test (CMT) score. Animals having SCC below 200,000 and showing CMT score of 0 and 1 were selected for collection of blood samples. Blood was collected from four animals. PBMC was isolated from blood at room temperature by density gradient centrifugation (Histopaque - 1.083 g/ml, Sigma, Poole, Dorset, UK). The isolated cells were suspended in red blood cell (RBC) lysis buffer so that lysis of any RBCs left during the separation procedure could be completed. Roswell Park Memorial Institute-1640 media, supplemented with 10% fetal calf serum, 0.1 mg/ml ampicillin, 0.1 mg/ml of kanamycin, 0.001 mg/ml amphotericin B, was then used to wash the isolated mononuclear cells by first mixing with the media and then centrifuging it at 200 g for 10 min each. Each time supernatant separated by centrifugation was thrown, and the pellet was kept for further processing. The recovery and viability of the isolated PBMC were determined by Countess™ Automated Cell Counter (Invitrogen, USA). 10 μl of 0.4% trypan blue was pipetted and mixed with 10 μl of cell suspension, which was incubated for 3 min at room temperature. Out of this mixture, 10 μl was then loaded in the chamber of Countess^®^ Cell Counting Slides (Invitrogen, USA). The slides were then inserted into the slide port of the countess, and the reading was taken. The total number of viable cells counted on an average was 1 × 10^6^/ml, accounting for more than 85% viability. Cells from each animal were then divided into four wells and were plated at a density of 1 × 10^6^ cells/ml. The PBMC was then challenged with PGN+LTA obtained from *S. aureus* cell wall (Sigma-Aldrich, Saint Louis, USA) at a dose rate of 10+10, 20+20 and 30+30 μg/ml of culture media for a total of 6 h in 6 well plates (3 × 10^6^ cells/well) at 37°C with 5% CO_2_. A non-challenged PBMC was kept as a control which was also maintained at 37°C with 5% CO_2_. The above process was again repeated on the 2^nd^ day for the same set of animals and the PBMC of the same animals and conditions (challenged and non-challenged) obtained by centrifugation after the final step of incubation was pooled together.

### Bovine PBMC challenge with PGN and LTA

The dose rate for challenge of bovine PBMC was decided on the basis of previously described protocols involving *in vitro* stimulation of bovine mammary epithelial cells or bovine PBMC by either LTA or PGN or a combination of both [13-15]. The time period of 6 h was considered because most of the inflammatory cytokines after reaching an expression peak at about 4-6 h were either found to have no change or showed a decrease in the level of cytokines even after 12 or 24 h of challenge [13].

### Total RNA extraction and quantitative real time-polymerase chain reaction (qRT-PCR) analysis

The procedure for RNA isolation was followed using RNeasy Plus Mini Kit (Qiagen) as per the manufacturer’s instructions. RNA Quantification was done by NanoDrop ND 1000 Spectrophotometer (Thermo Scientific, Wilmington, DE, USA). First strand cDNA synthesis was done using High-Capacity RNA-to-cDNA™ Kit (Applied Biosystems, USA) following the manufacturer’s instructions under the following conditions: 37°C for 60 min followed by 95°C for 5 min and subsequent hold at 4°C. Taking efficiency of cDNA synthesis as 100%, it was diluted to a final concentration of 5 ng/μl. Real-time PCR reactions were performed using Fast SYBR^®^ Green Master mix (Applied Biosystems, Warrington, UK). The thermal cycling program of the qPCR included one cycle at 95°C for 10 min followed by 40 cycles at 95°C for 15 s and 60°C for 1 min. A dissociation step was also included to confirm the specificity of amplification. Four biological samples and three technical replicates of each sample were used in this study. Comparative C_T_ method [16] was used to analyze the generated data.

The production of the melt curve as a result of the dissociation was observed for the presence of a single peak, which represented a single PCR product for each gene amplified. A negative template control was included in the RT-PCR reaction where instead of cDNA nuclease free water was added to the reaction mixture. For all sets of primers, reaction efficiencies were calculated using a two-fold serial dilution of pooled cDNA. A standard curve was constructed by plotting average CTs against log_10_ (cDNA concentration/100). The line obtained from the slope (m) was used to calculate the efficiencies of primers using the formula 10^(−1/m)−1^. Primers with efficiencies between 0.8 and 1.1 were considered acceptable and were included in the study (Table-1). Expression of β-actin was the most stable across all sample types (M value=0.55). β-actin was therefore selected as a reference gene for normalization of expression data. Log_2_ fold change conversion was made to the qRT-PCR data relative to control untreated samples using the 2^−ΔΔCT^ method.

**Table-1:** Sequences of oligonucleotide primers^#^.

Gene name	Primer sequence (5’-3’)	Amplicon size (bp)	Accession number	References
IL-10	F: CTTGTCGGAAATGATCCAGT R: TCTCTTGGAGCTCACTGAAG	208	NM_174088	Strandberg *et al*., 2005
IFNG	F: GTGGGCCTCTCTTCTCAGAA R: GATCATCCACCGGAATTTGA	234	M29867	Strandberg *et al.*, 2005
TNF-α	F: CTGGTTCAGACACTCAGGTCCT R: GAGGTAAAGCCCGTCAGCA	183	AF011926	Strandberg *et al.*, 2005
TLR-2	F: AGCACTTCAACCCTCCCTTT R: GAATCAGAATGGCAGCATCA	216	NM_174197	Strandberg *et al.,* 2005
IL-8	F: CACTGTGAAAATTCAGAAATCATTGTTA R: CTTCACAAATACCTGCACAACCTT	103	S82598.1	
GAPDH	F: TGACCCCTTCATTGACCTTC R: GATCTCGCTCCTGGAAGATG	143	BC102589.1	
HPRT	F: GTGGGATATGCCCTTGACTATAA R: GTGGGATATGCCCTTGACTATAA	104	NM_001034035.2	
β-actin	F: CATCGGCAATGAGCGGTTC R: ACAGCACCGTGTTGGCGTAG	146	BC142413.1	

# Primers for IL-8, GAPDH, HPRT and β-actin were designed using integrated DNA technologies online software PrimerQuest Tool and were verified by OligoAnalyzer Tool (Coralville, Iowa, U.S.A). IL-8=Interleukin 8, GAPDH=Glyceraldehyde-3-phosphate dehydrogenase, HPRT=Hypoxanthine phosphoribosyltransferase, TLR-2=Toll like receptor 2, TNF-α=Tumor necrosis factor-alpha, IFNG=Interferon gamma

## Results

### Cytokine mRNA expression of bovine PBMC against PGN+LTA challenge

**Figure 1:**
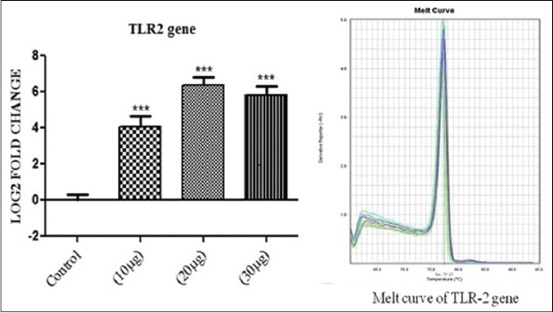
The figure depicts 4.06, 6.36 and 5.83 log_2_ fold highly significant difference of toll like receptor 2 mRNA expression n=4 in 10+10 μg, 20+20 μg and 30+30 μg peptidoglycan + lipoteichoic acid per ml of peripheral blood mononuclear cell challenged groups, respectively, as compared to control. Data are normalized to the reference gene. The one-way ANOVA with Newman-Keuls multiple comparison test was performed to determine the significant differences between ΔCTs of the analyzed groups.

**Figure 2:**
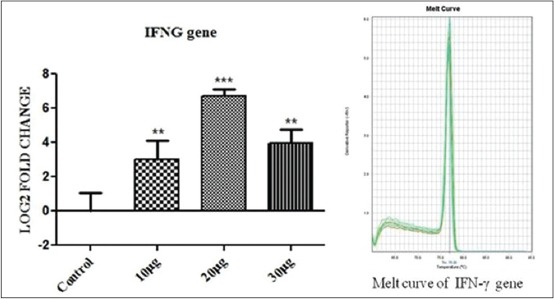
The figure depicts 3.03, 6.72 and 3.97 log_2_ fold significant up-regulation of interferon gamma mRNA expression n=4 in 10+10 μg, 20+20 μg and 30+30 μg peptidoglycan + lipoteichoic acid per ml of peripheral blood mononuclear cell challenged groups respectively, as compared to control. Data are normalized to the reference gene. The one-way ANOVA with Newman-Keuls multiple comparison test was performed to determine the significant differences between ΔCTs of the analyzed groups.

The effect of *S. aureus* cell wall antigens (PGN+LTA) at a dose rate of 10+10, 20+20, 30+30 μg/ml on bovine PBMC cytokine mRNA expression was determined in culture after 6 h of incubation. A control was also kept (bovine PBMC without any antigen) for 6 h of incubation for comparing the cytokine profiles of challenged samples. The result showed significant up-regulation of both TLR-2 and interferon gamma (IFNG) at all the doses of PGN and LTA combinations. The expression of TLR-2 gene showed a gradual increase in mRNA expression (p<0.001) with increase in the dose of the antigens 20+20 μg/ml followed by decrease with further increase in the concentration of the antigen blend 30+30 μg/ml, which has been shown in Figure-1. Similar fold change expression was also shown by IFNG, but the pattern was much steeper than that of TLR-2 mRNA expression profile. A significant up-regulation of IFNG (p<0.01) was observed at 10+10 and 30+30 μg/ml of *S. aureus* antigens (Figure-2). The up-regulation of IFNG was much more at 20+20 μg/ml antigen challenge (p<0.001).

**Figure 3:**
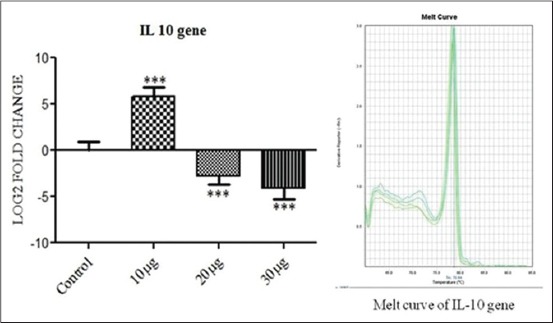
The figure depicts 5.77, −2.74 and −4.12 log_2_ fold significant difference of interleukin 10 mRNA expression n=4 in 10+10 μg, 20+20 μg and 30+30 μg peptidoglycan + lipoteichoic acid per ml of peripheral blood mononuclear cell challenged groups, respectively, as compared to control. Data are normalized to the reference gene. The one-way ANOVA with Newman-Keuls multiple comparison test was performed to determine the significant differences between ΔCTs of the analyzed groups.

**Figure 4:**
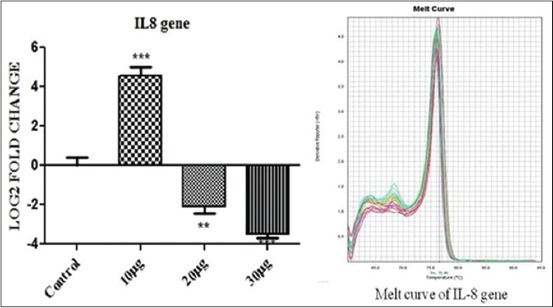
The figure depicts 4.56, −2.1 and −3.49 log_2_ fold significant difference of interleukin 10 mRNA expression n=4 in 10+10 μg, 20+20 μg, and 30+30 μg peptidoglycan + lipoteichoic acid per ml of peripheral blood mononuclear cell challenged groups respectively, as compared to control. Data are normalized to the reference gene. The one-way ANOVA with Newman-Keuls multiple comparison test was performed to determine the significant differences between ΔCTs of the analyzed groups.

**Figure 5:**
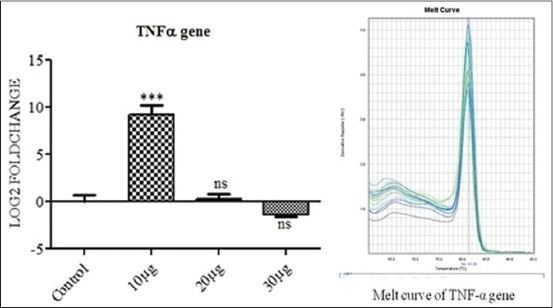
The figure depicts 9.22, 0.36 and −1.37 log_2_ fold difference of tumor necrosis factor-alpha mRNA expression n=4 in 10+10 μg, 20+20 μg and 30+30 μg peptidoglycan + lipoteichoic acid per ml of peripheral blood mononuclear cell challenged groups respectively, as compared to control. Data are normalized to the reference gene. The one-way ANOVA with Newman-Keuls multiple comparison test was performed to determine the significant differences between ΔCTs of the analyzed groups.

Interleukin 10 (IL-10) (Figure-3) and IL-8 (Figure-4) showed a significant up-regulation (p<0.001) when PBMC was incubated with 10 μg PGN with 10 μg LTA per ml of culture media. But as the dose was further increased to 20+20 μg and then to 30+30 μg antigen challenge per ml of culture down-regulation in the expression of the genes were observed at p<0.01 and p<0.001, respectively. Tumor necrosis factor-alpha (TNF-α) showed significant up-regulation of the expression at 10+10 μg of PGN+LTA challenge (p<0.001) but its expression became non-significant when the amount was increased to 20+20 μg of antigens per ml of media as shown in Figure-5. Moreover, TNF-α showed non-significant down-regulation with an increase in the quantity of antigen challenge (30 μg PGN + 30 μg LTA per ml of culture media).

## Discussion

Cattle with the higher response of cell-mediated immune response (CMIR) or antibody mediated immune response (AMIR) have lower incidence of mastitis in comparison to low or average responders [17-19]. Both CMIR and AMIR responses are produced by blood mononuclear cells. TLR signaling as well as cytokines, influence macrophages and dendritic cells to act as antigen presenting cells and thus, activate T-helper (Th) cell sub-populations. Th1 cells predominately produce cytokines that generate a CMIR in response to intracellular microbes [20]. In contrast, Th2 cells fabricate those cytokines that tend to generate an AMIR toward extracellular pathogens such as *E. coli* which is known to cause mastitis [20]. TNF-α, IFNG, and IL-8 release favors Th1 type response whereas IL-10 is biased toward Th2 type response of immune cells [21]. Th1 type response is specific and is of main importance for the development of modern vaccines. There is increase in the level of Th1 and Th2 type cells in mammary gland mediated by proinflammatory cytokines when infected by *S. aureus* causing mastitis; the release of proinflammatory cytokines is tightly regulated by the production of IL-10 cytokine [22]. In the United States, J5 vaccine for protection of mastitis against *E. coli* has been used successfully, which has been explained on the mechanism of increased antibody production and greater opsonization of lipopolysaccharides and the coliform bacteria [23].

At the site of infection, TNF-α promotes activation of cells and recruitment of leukocytes. TNF-α is also produced by monocytes, macrophages, and lymphocytes [24]. Sources of IFNG, IL-8, and IL-10 genes include lymphocytes and cells of monocytic lineages [25]. IFNG forms an integral component of the innate immune system and acts by enhancing the phagocytic activities of monocytes and macrophages. Increase in IL-8 activity is followed by recruitment of neutrophils and to lesser extent T-lymphocytes [26]. IL-10 as an anti-inflammatory cytokine is involved in the regulatory activity of pro-inflammatory cytokines [27].

The results demonstrate that TLR-2 acts as an important PRR which also affects or control the signaling of other important cytokines. Gram-positive bacteria cell wall components such as PGN and LTA activates immune cells, which is mediated by TLR-2 [28]. TLR-2 agonists PGN and LTA enhances IFNG release specific to the antigen in whole blood [5]. We demonstrated that PGN and LTA were able to induce similar responses in both TLR-2 and IFNG. With the increase in the dose of antigens, expression of both TLR-2 and IFNG elevated but followed by a decrease with more increase in the dosage of the antigens. This pattern gives an indication that the level of IFNG is influenced by the expression of TLR-2 gene. IFNG has been reported as an important feature of innate and adaptive immunity and is specified by the quick response and increased production against an infection [27]. The results of this study showed significant up-regulation of TNF-α at low doses of antigen stimulation of cattle PBMC which was similar to those found during *in vitro* study using either live or dead bacteria [28]. TNF-α stimulates endothelial activation and recruitment of leukocytes to the site of infection [29]. Besides, TNF-α was also proposed as one of the potential DNA markers in the improvement of immunity to mastitis [30].

IL-8 and IL-10 also showed significant up-regulation at low dose challenge which shifted towards down-regulation on stimulation of PBMC with higher doses. IL-8 gene was found to be up-regulated in blood mononuclear cells during mastitis caused by *S. aureus* [31]. Our results showed an increase in the quantity of IL-8 mRNA with rise in the level of TNF-α. However, the expression was down-regulated or decreased as the amount of cell wall antigen challenge was increased. IFNG, TNF-α, and IL-8 act as pro-inflammatory cytokine, whereas IL-10 act as anti-inflammatory cytokine. Anti-inflammatory effect on monocytes, macrophages and neutrophils is exerted by IL-10 by decreasing the production of chemokines, cytokines and eicosanoids involved in proinflammatory reactions [32]. IL-10 being an anti-inflammatory cytokine; its main function is to control the excessive release of certain pro-inflammatory cytokines like TNF-α [24]. The result indicates that IL-10 is involved in the regulatory mechanism of TNF-α release from immune cells present in PBMC which helps in the avoidance of TNF-α mediated septic shock. IL-10 being the most important anti-inflammatory cytokine also suppresses the expression of IFNG to shift the Th1 type response to Th2 type response [32]. But, as the dose of the antigens was increased in our study, the level of IL-10 did not increase to counter the effect of increase in IFNG expression. The result strongly suggests that the expression of IL-10 followed the expression of TNF-α and was not influenced by the expression pattern of IFNG. The overall decrease in the expression of genes at higher concentrations of antigen challenge in the current study might be due to cellular apoptosis induced by TLR-2.

The study indicates the potential of cell wall antigens of *S. aureus* to be used for the production of vaccines as these antigens are also able to activate immune responses in cell cultures. The study is also beneficial for *in vitro* challenge and comparison of innate immune response of different breeds of animals, as well as a comparison between different species, which may help in generating data giving an indication about the difference in the pattern and regularity of mastitis infection between different breeds and species of animal.

## Conclusion

It can be said that a dose of 10 μg of PGN and 10 μg of LTA per ml of culture medium was found to be most suitable for challenging PBMC. The mRNA expression of genes taken under study showed similarity to studies in which cells were challenged either by live or killed *S. aureus*. The research revealed about the involvement of TLR-2 activity as a major PRR with coordinated activities of cytokines involved in Th1 and Th2 types of immune response.

## Authors’ Contributions

BB, MP, PK and SS: Planning of the research and analysis of the result. SS, AV and NAB: Carried out all the research work (culture, challenge. RNA isolation, cDNA synthesis, qRT-PCR reactions, arranging data in excel). All authors participated in draft and revision and approved the final manuscript.
